# Elémens de Chimie

**Published:** 1836-07

**Authors:** 


Art. V.—*Elémens de Chimie.* Par E. Mitscherlich, Professeur de Chimie à Berlin, &c. *Traduits de l'Allemand sur la dernière Edition*, par M. B. Valerius, de l'Université de Gand.—*Bruxelles*, 1835-6. Tom. I. and II. 8vo. pp. 415, 296.

We were greatly interested by the announcement for publication of a Treatise on Chemistry by Professor Mitscherlich, of Berlin. His reputation as an able and popular teacher, his celebrity as an exact and philosophic chemist, and, above all, the estimation in which he is universally held as an original discoverer in some of the profoundest branches of the science, excited a curiosity, and raised in us expectations, which we have never in the same degree experienced upon any previous occasion. A rapid perusal of the first two volumes of his "Elémens," even though in the disadvantageous garb of a French translation, (not always, we fear, very faithfully made,) has more than justified the anticipations in which we ventured to indulge; and, gratified as we have been by the instructive and perspicuous manner in which he has handled topics of an elementary nature, we cannot but look forward with increased interest to the completion of a work which will disclose to us, in the words of the author, his matured views in reference to the doctrines of Isomorphism, or the relation which subsists between the chemical constitution and the crystalline forms of bodies.

In the preface prefixed to the second volume, the plan of the work is very clearly explained, and a brief account given of the contents of the different volumes, which, including the two already published, will altogether amount to five. Upon the unpublished volumes we are, of course, not in a predicament to make any critical observations, and shall therefore merely state that the third will be devoted to the metals, their combinations, and those compounds of organic origin important in a theoretical point of view; the fourth, to the doctrines of combination in definite proportions, and the discussion of those branches of mechanical science, such as Crystallography, Heat, Light, Electricity, and Magnetism, the knowledge of which is so essential to the modern chemist; while the fifth will present a recapitulation of the general results dispersed through the preceding volumes, a development of the relations of chemistry to the other sciences and the arts, and an abridged history of the successive steps by which it has reached to its present comparatively perfect state.

As respects the scope of his Elements, our author describes himself as composing a Manual for Students, not a System professing to include all the known facts of chemistry. Such an undertaking as the latter he justly considers as in a great measure superfluous, since the publication of the French edition of his great work by Berzelius. The plan which he pursues has been dictated, he alleges, by a desire to facilitate the business of instruction. It is materially different from that generally employed by British chemists, who, as is well known, before proceeding to the detail of chemical facts, are in the habit of giving a view of the general laws deduced from these facts, and to which reciprocally the facts may for explanation be referred. This synthetic method, alone admissible in the exacter sciences, is, he contends, inapplicable to chemistry; for "it is impossible that he who has not seen the phænomena can form a clear idea of the science itself, of the parts into which it is divided, and of the laws and general results to which the study of it conducts. Thus, one cannot form a clear conception, for example, of chemical affinity, of chemical combinations and decompositions, of the solid, the liquid, and the gaseous state of bodies, without first witnessing the phænomena from whence these facts are deduced. The method, therefore, of treating chemistry which is at once the most easy and the most perfect, consists in performing before the pupil experiments, by means of which he can create the science himself, and be rendered attentive to phænomena, to their explanation and connexion with each other, without ever building upon facts which he has neither seen and explained, or which he cannot see immediately."

In the general justice of these remarks there are, we believe, few who will not concur. But, while our author feels strongly, and expresses clearly, the objections to the mode usually pursued, he would appear to overlook, or at least to keep in the back-ground, difficulties of no trifling magnitude, by which the method he himself adopts is beset. If the chemical student be introduced at once to phænomena, with the view of his creating from them a science, he will, we fear, be frequently found incompetent to the task, and, after having loaded his memory with a mass of facts which to him appear without order, connexion, or explanation, will abandon in despair a study which has none of the attractions of a science, and would appear to require of its cultivator almost superhuman powers of memory. We have no hesitation in giving it as our decided opinion, founded on experience, that the purely analytic method here advocated is not that by which a knowledge of chemistry is most rapidly conveyed. We consider a brief explanation of the laws which regulate chemical combination to be an indispensable preliminary to the practical parts of the course; and, though a perfect demonstration of these laws to the mere beginner is a thing scarcely to be accomplished, should we succeed in impressing mere results upon his mind, his progress will be greatly facilitated; for, in the various phænomena with which he will subsequently be engaged, he will find but exemplifications and confirmations of some general principles with which he is already furnished. Our author himself, constrained (we presume) by the necessity of the case, commences his second volume with a brief exposition of the laws which govern the combination of bodies, both by volume and by weight, and thus bears, as it were, unwilling testimony in favour of the method against which he has so ably argued.

The first of the volumes before us includes the history of the non-metallic elements, and of the various compounds which result from their mutual union, with the exception of those possessed of an acid nature, and concludes with two very able and original articles (as far as respects the manner in which the subjects are handled,) upon Atmospheric Air and Water, under both of which a variety of the most interesting discussions are introduced. In the second the subject of the ordinary acids is examined, under the head of the Oxacids and Hydracids; the former being arranged according as they include a simple or a compound radicle; after which occurs a succinct, but at the same time very clear, account of a variety of other compounds constituted in a very different manner, but possessed of the same electro-negative virtues. If we omit some obvious errors of the press or the translator, there is scarcely any thing in these volumes which does not meet with our unqualified approbation. The experiments are simple, conclusive, and described with such remarkable clearness and precision as to admit of easy repetition by the student. The apparatus also employed, even when of the least complicated kind, is always illustrated by a plate; a circumstance of the utmost importance to him who, unable to attend in the laboratory of an analytic chemist, is compelled to derive all his information from books. But the characteristic feature of the work, and that which confers upon it its highest value, is the detailed exposition it contains both of the physical and chemical phænomena which present themselves in each experiment, and the frequent incidental application of the demonstrated laws of chemistry to the explanation of various phænomena and processes in manufactures and the arts. This union of chemical and mechanical science renders the knowledge conveyed as complete as possible; while an occasional exemplification of their value in the creation of individual and national wealth cannot fail to awaken the curiosity and sustain the interest of those who do not value science so much for its own sake as for the important practical purposes to which it may be turned.

We have, however, not exhausted our commendations of the Elémens of Professor Mitscherlich. His work is not a mere well-digested summary of the generally known facts and theories of chemistry: it includes the results of recent very important researches of his own, which, though they may have appeared in some of the continental periodicals, are but very imperfectly known in this country, not having as yet found their way into our class-books and standard works. Of this description are, in particular, his observations on the theory of the production of æther. This remarkable fluid, it is well known, is usually obtained by distilling a mixture of equal weights of oil of vitriol and rectified spirit, and is purified by agitation with water containing a little caustic potash, and rectification from dry chloride of calcium. Now, chemical analysis having long since established that æther may be represented by $C^4H^4 + H^1$, and alcohol by $C^4H^4 + 2H^1$, it was natural to conclude that the oil of vitriol, in virtue of its affinity for water, deprived the alcohol of $1 H^1$, and thus converted it into æther. This was accordingly the theory first proposed, and it was for a considerable time generally adopted. When, however, it was ascertained that the spirit and oil of vitriol, when mixed, gave rise to a peculiar acid, (the sulphurinic,) and that, during the distillation of the æther, two other products are formed, the heavy and the light oil of wine, this simple theory obviously required modification. A modification it has accordingly undergone;* but, when this is accurately considered, the function of the oil of vitriol, as far as respects the development of æther, is still in effect considered to be the removal of water from the alcohol. Now, these views are contested by our author, on the following conclusive grounds: 1. That acid containing two atoms of water is more efficacious than oil of vitriol, which contains but one. 2. That, when absolute alcohol is brought in a continuous stream in contact with such acid, its influx being so regulated that the temperature may continue permanently at 282°, it is almost entirely converted into æther; and with this there distils over the exact amount of water which the alcohol must have lost in the process. These facts require little comment: they fully disprove the common theory; for, if this were true, the acid should be efficacious in proportion to its strength, and the water extracted from the alcohol, instead of passing off with the æther, should obviously be detained in the still or retort.

But, if sulphuric acid does not act by its affinity for water, what is its *modus operandi?* To this interrogatory Mitscherlich replies, that it is a case of *decomposition by contact;* or that, as peroxide of manganese resolves peroxide of hydrogen into water and hydrogen gas, so alcohol, in the presence of oil of vitriol, is decomposed into water and æther, the acid at the same time undergoing no change whatever.

Such is a brief sketch of one very original and valuable passage in the first volume; and several others of a similar character will be detected by the attentive reader. In fact, one of the great merits of the work would appear to consist in the circumstance of every disputed topic having been submitted by the eminent author to a rigorous experimental examination. Ingenious and novel methods of analysis are everywhere interspersed throughout the work, and, by means of their chemical problems, are satisfactorily solved, (*ex. gr.* the composition of hyponitrous acid,) which are altogether untouched by ordinary chemical writers.

We conclude these observations by strongly recommending Professor Mitscherlich's book, not only to students but to adepts in the science; and expressing a conviction, arising from the specimen before us, that, when completed, it will constitute one of the most instructive and fascinating digests of chemical knowledge which has as yet been presented to the public.

* Berzelius, Traité de Chimie, tome vi. p. 494.

